# Heavy Metal Content in PolyfloralHoney and Potential Health Risk. A Case Study of Copșa Mică, Romania

**DOI:** 10.3390/ijerph17051507

**Published:** 2020-02-26

**Authors:** Szilárd Bartha, Ioan Taut, Győző Goji, Ioana Andra Vlad, Florin Dinulică

**Affiliations:** 1 Department of Forestry and Forest Engineering, University of Oradea, Gen. Magheru Street nr. 26, 410048 Oradea, Romania; barthaszilard10@yahoo.com; 2 National Institute for Research and Development in Forestry “Marin Drăcea” S.C.D.E.P., 400202 Cluj, Romania; 3 University of Agricultural Sciences and Veterinary Medicine, 400372 Cluj-Napoca, Romania; 4 Technological High School Ştefan Manciulea, 515400 Blaj, Romania; ggyozo2000@yahoo.com; 5 Department of Food Engineering, University of Oradea, Gen. Magheru Street nr. 26, 410048 Oradea, Romania; ioana_andravlad@yahoo.co.uk; 6 Department of Forest Engineering, Transilvania University of Brașov, Sirul Beethoven Street nr.1, 500123 Brașov, Romania; dinulica@unitbv.ro

**Keywords:** health risk, AAS (atomic absorbtion spectrometry), historical polluted area

## Abstract

Honey is both a complex food and medicine as well as a healthy alternative to refined sugar. Besides a complex mixture of carbohydrates, honey contains other minor substances which may threaten human health in excess concentrations. Several environmental conditions can affect the quality of honey. This research paper aims to measure the degree of heavy metals (Lead (Pb), Cadmium (Cd), Zinc (Zn), and Copper (Cu)) in some polyfloral honey from an industrial area of Romania, considered to be one of the most polluted regions in Eastern Europe. The samples were collected from six stationary apiaries and analysed using the atomic absorption spectrometry method. The content of Pb was higher in the sampling areas exposed directly to the polluted air masses. Cd concentration decreases exponentially while Cu concentration increases as the distance from the source of pollution increases. The checking of the quality of polyfloral honey from local producers is imperative because this product is intended to be consumed by the beekeeper’s family or the local community without being sold to an authorised processor. The results of the study can help to set a threshold for the concentration of Pb and Cd in honey marketed in the European Union.

## 1. Introduction

Food safety has become an essential food quality attribute, not only because of the major role played by foodstuffs within a human healthy diet, but also the issue of public concern [1]. The presence of heavy metals in foodstuffs is becoming increasingly more obvious and food consumption represents the main way of access to the human body [1,2] with potentially negative effects on human health, especially because heavy metals de-regulate the immune system [3] and lead to severe diseases, including cancer, cardiovascular diseases, and neurological disorders [4,5,6]. Heavy metals persist and biomagnify in trophic chains, and when in concentrations above the maximum permissible limits, they become toxic [7]. High concentrations of heavy metals pose a risk to consumer health, while prolonged consumption of honey containing Cu and Fe causes gastrointestinal disorders [8]. Heavy metals can affect the quality of life when these are accumulated in the body at a toxic level, and may threaten the health of consumers [9,10].

Honey represents an important element of the human diet because of its positive nutritional and health effects [11]. Honey is a bee product from the nectar or excretions of plants, composed of a complex mixture of carbohydrates [12]. The main nutritional value of honey is due to the presence of simple, inverted sugars, such as glucose and fructose, which are an immediate and prompt source of energy for the human body—for example, 100 g of honey generates 300 kcal of energy [13]. According to Bilsel et al. [14], honey displays its curative properties in the treatment of diabetic ulcer and external wounds in the case of allergies, inflammation of the pharynx, and coughs, and it also has visible antimicrobial properties [15], can prevent gastroesophageal reflux [16], and its components have shown apoptotic effects in colon cancer cells [17]. Honey contains minor constituents, such as enzymes, proteins, amino and organic acids, vitamins, lipids, volatile chemicals, flavonoids, phenolic acids, and minerals [18,19,20]. The quality and biochemical proprieties of honey depend on the nectar source, climatic conditions, maturation period of the honey, production methods, and processing and storage conditions. Honey also contains macro- and microelements that are the minor constituents of honey present in the range of 0.02%–1.03%. The heavy metal concentration in honey depends on the geographical origin of the flower composition [21] and its presence indicates that the hives were close to contamination sources. The concentration of heavy metals is correlated with the honey’s spatial distribution from the main pollution source, but also with the air dispersion conditions from the area [22].

Hernandez et al. [23] list K, P, Mg, Al, Ca, Na, Fe, Mn, Cu, Zn, Cl, S, and Si as common mineral components in honey, while Batista et al. [24] note that potentially toxic metals, such as Pb, Hg, and Cd appear at lower concentrations than other heavy metals.

Porrini et al. [25] showed the concentrating role of bees regarding heavy metal pollutant accumulation in bee products, highlighting that the hair on the bee’s body retained atmospheric particles containing heavy metals. Anthropogenic sources can also contaminate honey [26].Historical pollution of the environment contributes to the more pronounced internalization of pollutants in the hive [27,28]. High levels of Cd in honey were detected in agroecosystems due to the use of mineral fertilisers and pesticides [29]. Regardless of the polluting source, both Pb and Cd are heavy metals that contaminate bee products [30].

Setting up the apiary in the vicinity of a point source or of diffuse pollution sources, the use of agrochemicals, fertilisers containing heavy metals, and the fumigation of bees causes the contamination of honey and bee products and the intake of pollutants into the hive [31]. Gonzalez et al. [32] explained that high Cd content in honey could arise by improper storage or equipment-related contamination. Contamination of honey and other bee products may be due to the environment or improper storage, handling, or processing. Storage in containers and the use of galvanised tools can also contaminate honey with Zn [33].

Bees collect pollutants from the environment, such as radioactive substances, heavy metals, and inorganic compounds, with all of these being indicators of local pollution. Perugini et al. [34] considered honey to be a time and spatial Pb contamination detector. By visiting several flowers, beesbioconcentrate metal pollutants from the environment, which can have potentially harmful effects on human health. According to Celli and Porrini [35], the flying range of bees covers an area of about 7 km^2^, and as the bee gets in direct contact with heavy metals deposited on plants, the honey and other bee products become a good indicator of the degree of environmental contamination [30,36].

According to European Committee (EC) [37], Romania is one of many honey-producing countries, alongside Spain, Hungary, Germany, Italy, Greece, France, and Poland which are all countries with a favourable climate for beekeeping. In this context, studies were conducted in order to determine the quality and volatile compounds of Romanian honey [38,39,40]. In 2015, honey production in Romania was 35,000 tons. Roman [26] showed that the honey market was imposing ever-higher quality standards, where consumers are asking for quality honey with no additional waste nor loads of potentially toxic metal pollutants. Due to an increase in the anthropological footprint of ecosystems and the degree of pollution thereof, the quality of honey is an obvious and stringent issue which concerns both honey producers and processors, where appropriate, and especially consumers who consume unprocessed honey directly from producers. 

The purpose of this research is to determine heavy metal content (Pb, Cd, Cu, and Zn) in honey from private apiaries located in the most well-known historical polluted area in Romania. The analytical values obtained can provide information on the regional dynamics of pollution and allow for the adoption of acceptable thresholds in terms of the heavy metal content of natural honey, which are currently not in place.

## 2. Materials and Methods

### 2.1. Sample Collection Area

**Figure 1:**
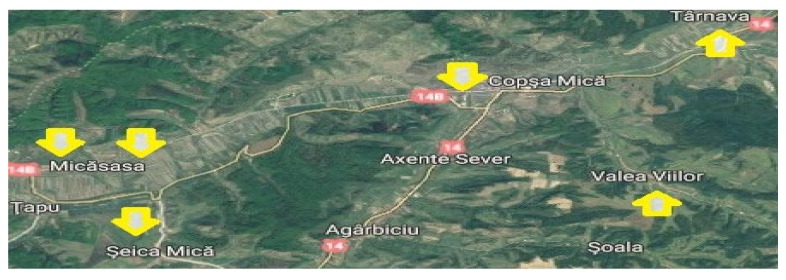
Map of the honey sampling localities.

The polyfloral honey samples were collected directly from local beekeepers at six stationary apiaries (four samples from each apiary) from the town of Copșa Mică, Romania and the neighboring villages of Micăsasa, Târnava, Valea Viilor, and Şeica Mică (Table 1, Figure 1). 

This paper aims to identify the presence of potentially toxic heavy metals in the honey collected from an environment polluted for over 60 years by industrial activity with non-ferrous metals on the industrial platform of Copșa Mică. The dispersion of pollutants emitted by the main polluter of the Copșa Mică area is governed by the local climate. The wind regime of the area is influenced by the land orography, especially the presence of the Târnava Mare valley, the main air masses being channeled into the corridor of the Târnava Mare river (northeastern and southwestern air circulation) and the corridor of the Visa river (South–North air circulation).

The source of the pollution whose effects are analyzed in this article is an industrial park in the town of Copșa Mică (46°06′59.10″ N and 24°13′15.43″ E) in the center of Romania.

### 2.2. Method of Analysis

The collected samples were stored in hermetically sealed plastic containers and kept in a cool and dark space at temperatures ranging between 4–5 °C until analytical determinations were carried out. Before analysing the polyfloral honey samples without visible granules, the samples were shaken for homogenization purposes, and those containing crystallised sugar (four samples) were heated to 65 °C in a water bath for 30 minutes to homogenise and solubilise the crystals. From the homogenised samples, 1 g of honey was weighed into polypropylene tubes, which were dissolved in 100 ml of heated, deionised, ultra-pure water(18.2 MΩ-cm resistivity from the water purification system Direct Q3UV Smart, Milipore, SAS, Molsheim, France). The solutions thus obtained were subjected to wet mineralisation, using 0.5 ml of analytically pure HNO_3_ (Merck, Darmstadt, Germany) concentrate (65% v/v) as an oxidising agent, and the Top Wave Analytic Jena AG (Germany) microwave system was also used, designed for pressure digestion at temperatures up to 230 °C and pressures up to a maximum of 100 bars (1450 psi). The digester could contain a maximum of 24 digestion vessels made of modified polytetrafluorethylene (TFM-PTFE). Vessels were cleaned with 50 ml HNO_3_ before each mineralization. The glassware used was left overnight in a 10% HNO_3_ solution and rinsed prior to use with purified water to prevent possible contamination with Pb, Cd, Cu, and Zn. The extract was then filtered, ransferred to 25 mL volumetric flasks, and made to volume with deionised water. Mineralization was carried out in four steps, at temperatures of 145, 170,190, and 100 °C (Table 2).

Digestions of polyfloral honey samples were carried out in triplicate. A blank digest was carried out.

For the quantitative analysis of Pb, Cd, Zn, and Cu in the obtained extracts, we used the atomic absorption spectrometry method (AAS) because the harvested honey originated from a medium historically affected with a significant load of metal pollutants [41]. Quantitative determination of Cu and Zn was realized by FAAS (flame atomic absorbtion spectrometry) [42] while the concentration of non-essential trace metals Pb and Cd was performed using GFAAS (graphite furnace atomic absorption spectrometry) [43]. All samples were analysed in triplicate, and mean values were obtained. We used the Shimadzu AA-6300 Spectrometer equipped with a graphite furnace and single-element hollow cathode lamps adapted to eachanalysed metal (Cu was measured using the multi-element hollow method lamp), and the D2 lamp for background correction (BGC-D2). In FAAS for determination of Zn and Cu, an air-acetylene flame was used (Acetylene purity 98%, flow rate 1.8–2.0 L/min.) and a slot burner head from titan which was 10 cm long. For graphite furnace determination, argon (Ar) was used (flow rate 0–1.5L/min). The instrument parameters were optimised following the manufacturer’s recommendations (Table 3). Merck standard solutions (1000 mgL^−1^) of Pb, Cd, Zn, and Cu were used to prepare the working standards [44]

During the analyses, the argon flow rate through the graphite tube was 250 mL/min. Settings for graphite furnace atomic absorption spectrometry (GFAAS) are summarized in Table 4.

## 3. Results

All samples subjected to analytical determinations had detectable Pb, Cd, Cu, and Zn content. Concentration values show a significant dispersion; the differences between the apiaries were statistically less reliable for copper (Table 5). Statistical analyses was performed using STATISTICA 8.0 (Stat Soft, Tulsa, OK, USA). 

The order of the accumulation degree expressed in median concentration values mg/kg for the elements surveyed is Zn > Cu > Cd > Pb.

The differences in terms of frontal exposure versus secondary valley exposure are statistically significant only for the Pb content (Table 6), which is, on average, 1 mg/kg higher in the apiaries located on the main valley, which is the driver for the pollutants of the apiaries on the side valleys.

**Figure 2:**
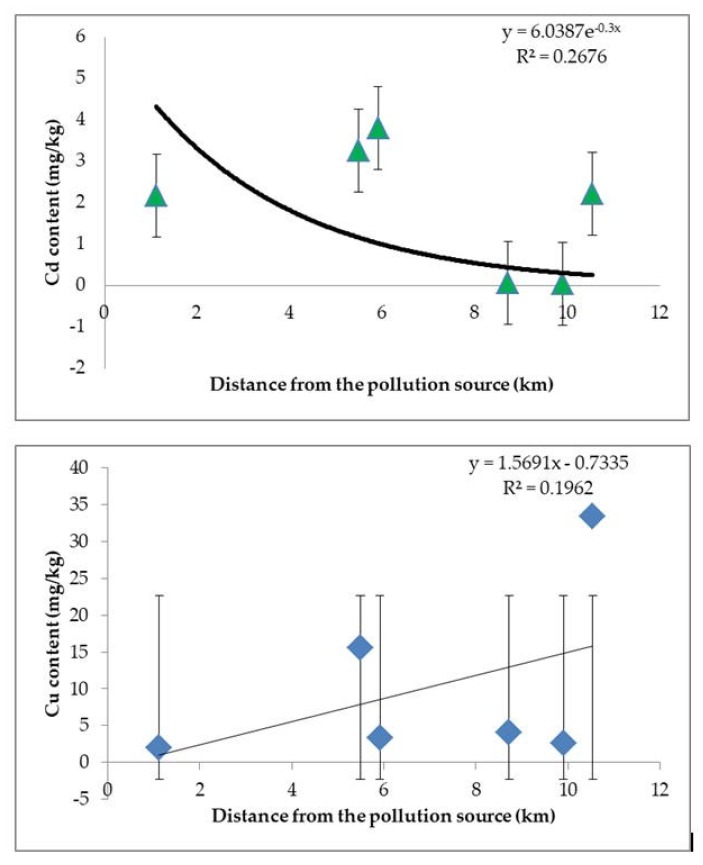
Cd (up) and Cu (down) content in honey against the distance from the main source of pollution.

From the Figure, concentrations of Cd in honey show a decreasing trend while moving away from the source of pollution, while Cu concentrations are increasing (Figure 2). Levels of Pb and Zn vary independently of the distance from the source of pollution (Spearman rank-order correlation = −0.029, *p* = 0.96).

## 4. Discussion

The polluters on the industrial platform were present mainly in the valley of the Târnava Mare river being scattered to the West (village of Micăsasa and city of Blaj), the East (city of Mediaş and town of Dumbrăveni), or South in the direction of the Șomârd-Şoala villages [45]. The preferential circulation of the air masses guided by the orography of the land resulted in the accumulation of significant amounts of Pb and Cd in the honey sampled from the localities of Târnava (located east of the main polluter) and Valea Viilor (southeast of Copşa Mică). The analytical values of Zn and Cu are above those determined in the honey sampled in the town of Copşa Mică, which supports the model of the remote dispersion of pollutants and the importance of the role of orography.

The accumulation of Pb in the environment is mainly due to mining, the melting and production of metals, and the battery industry, as Pb is a non-essential metal that has no physiological role in the metabolism of plants or animals. The exposure to Pb occurs through contact with soil, air, water, and contaminated food [46], and in time it causes anemia, neurological effects, nephropathy, renal tubular dysfunction, as well as impairment of reproductive function in both genders [47].

Although it is easily surpassed by Cd in terms of average and maximum levels, the honey sampled contained significant amounts of concentrated Pb (Table 2). Golob et al. [48] determined a maximum Pb concentration of 79.1 mg/kg in honey sampled in Slovenia, and Yilmaz and Yavuz [49], discovered high values of 4.2–6.3 mg/kg Pb in honey sampled in the Southeast Anatolia Region, Turkey. Concentrations of up to 80.37 mg/kg were detected in honey sampled in the polluted areas of Italy by Dambrosio and Marchesini [50]. Frias et al. [51] determined high Pb concentrations of 31.50 mg/kg in the honey sampled in Tenerife, Spain. The maximum Pb concentrations are lower than those obtained between 2005–2011 by Berinde and Michnea [52], who also determined Pbconcentration values ranging between 0.18–20.34 mg/kg in the area of the city of Baia Mare, which is also a historical polluted area of Romania. Bogdanov [30] reviewed the results presented in various papers in the literature and noted concentrations of Pb in honey ranging between 0.01–1.8 mg/kg and concentrations of Cd ranging between 0.03–2.1 mg/kg.

As far as the Pb content in Polish honey goes, Pb concentration values above 1 mg/kg were also obtained by Roman [27] from the honey sampled in the Legnica and Glogow industrial regions (Pb median values ranging between 0.465–1.097 mg/kg) and between 0.17–1.90 mg/kg in the Wroclaw region (Lower Silesia), [53]. Oddi and Bertani [54], Conti et al. [55], and Porrini et al. [28] determined maximum Pb concentration values ranging between 1.10–1.74 mg/kg in polyfloral honey sampled in Italy. Singh et al. [56], reported Pb concentration values ranging between 0.2–4.2 mg/kg in the case of 13 samples of monofloral and polyfloral honey collected from different regions of Karnataka, India. An analysis of some honey batches collected on the Czech market in 1999 from different honey producers indicated Pb concentration values ranging between 0.0184–1.0003 mg/kg [57].

Low concentrations of Pb which are below the values presented in our research were discovered in honey samples studied by Tuzen and Soylak [26] in Central Anatolia, with values ranging between 0.0176–0.0321 mg/kg; the same goes for Matusevicius et al. [58] in samples from different areas of Lithuania, being 0.0032–0.0241 mg/kg; Derebasi et al. [59] determined very low concentrations of Pb (6.68–7.30 ppb/kg) in honey sampled directly from producers in the Black Sea Region of Turkey in 2007, and Dzugan et al. [60] mentioned in their research maximum levels of Pb of 0.18 mg/kg in polyfloral honey sampled from Podkarpackie in Southeast Poland. In Romania, Simedru et al. [61] also determined low Pb concentration values (0.06–0.19 mg/kg) in honey sampled in Cluj County.

With regard to Cd concentrations, similar or lower levels in comparison with those determined in our research were reported by Dzugan et al. [60] (0.03 ppm) in Podcapacia, Southeast Poland; Derebasi et al. [59] (0.07 mg/kg) in Turkey; Tuzen and Soylak [26] (between 0.010–0.022 mg/kg) in Central Anatolia-Turkey; and Celechovska and Vorlova [57] (between 0.0005–0.0774 mg/kg) and Singh et al. [56] (0.005–0.76 mg/kg) in Karnataka India. Matusevicius et al. [58] reported maximum Cd concentrations (between 0.0039–0.0165 mg/kg) in different regions of Lithuania. Cd concentration values in polyfloral honey in the sample plot of Târnava, Micăsasa II, CopşaMică, and ValeaViilor surpassed the ones obtained by Bratu and Georgescu [62] in CopşaMică at a distance of 8–25 km from the main source of pollution. Polyfloral honey from the sample plot of ŞeicaMică and Micăsasa I was found to have a lower Cd content, as against the one analysed by Bratua nd Georgescu [62] (0.015–0.032 mg/kg).

The maximum concentration of Cd of 3.809 mg/kg that was determined from the polyfloral honey sampled from Târnava apiary was below the values observed by Frias et al. [51] (46.32 mg/kg Cd) in Tenerife, Spain. Simedru et al. [61] showed that the level of Cd in honey samples in Cluj County was below the detection limit of the used equipment. Devillers et al. [63] studied 150 samples of acacia honey from polluted areas (50%) and “virtually unpolluted” areas in the territory of France, and noticed the absence of Cd and Pb from the analysed samples, although other metals such as Ag, Zn, and Cr were detected as consequences of anthropogenic pollution.

Although zinc is an essential element, excessive bio-accumulated concentrations are the effect of anthropogenic intake. Acute poisoning with Zn occurs through inhalation at the workplace with symptoms such as nausea, vomiting, diarrhea, lethargy, and fever [64].

The honey examined by us contains the essential Zn and Cu trace elements in concentrations ranging between 15.00–36.40 mg/kg in the case of Zn, the median value being 20.400 mg/kg, and between 2.00–33.00 mg/kg in the case of Cu with a median value of 3.70 mg/kg. The maximum values close to the Zn values determined by us are those reported by Celechovska and Vorlova [57], as well as by Tuzen and Soylak [26] (Table 7).

The level of heavy metals in honey can often be increased by poor harvesting and storage conditions, and contamination during the fumigation, extraction, and storage of honey which are non-compliant with hygiene standards recommended or required by law. This is why honey monitoring is required at both the producer and processor levels. The high values in terms of non-essential heavy metals concentration measured in sampled honey are a warning for the local population, as in terms of the provisional tolerable weekly intake (PTWI) for Pb and Cd, other food coming from a polluted environment also participates, and the potentially toxic heavy metal concentration in the human body subsequently increases.

According to EC 2000a, Regulation (EC) No. 396/2005 [66], Food Standard Agency, 2003: The Honey Regulations 2003 [67], the current legislation does not mention maximum permitted limits of heavy metals in honey. However, according to Byrne [68], the European Commission recommends an acceptable maximum level of 1 mg/kg in the case of Pb and 0.1 mg/kg in the case of Cd.

Commission Regulation (EC) No. 1881/2006 of 19 December 2006, in setting maximum levels of contaminants in foodstuffs [69] (as amended by the Commission Regulation (EU) 2015/1005 of 25 June 2015 [70] sets the maximum level of Pb at 0.10 mg/kg and of Cd at 0.05 mg/kg. Thus, as to the median value of Pb, the content of honey harvested in Copşa Mică and its surroundings exceeds 14.9 times, and the median value of Cd exceeds 44 times the maximum permissible limits imposed by the European Commission in foodstuffs. Two samples of honey were from Şeica Mică and Valea Viilor, respectively; the villages of Şeica Mică and Micăsasa I had concentrations of Pb and Cd below the maximum permissible limits. Both maximum and median concentrations of Zn and Cu pose no risk of toxicity to the human body.

## 5. Conclusions

Historical pollution in the surveyed area, totaling over six decades of permanent contact with pollutants, marked the main bee product—honey. The results of this study offers valuable information regarding the effects of the environment on the quality of polyfloral honey produced in the Copșa Mică area. Analytical determinations of the content of potentially toxic metal pollutants in the polyfloral honey collected from the local bee producers with stationary hives in the closing year of industrial production activity indicated high concentrations of accumulated heavy metals, posing a risk to the health of consumers. The apiaries located on the valley that channel the pollutants from the industrial platform are prone to higher Pb bioaccumulations than the apiaries situated on the side valleys. Concentrations of Cd were exponentially diminishing while moving away from the source of pollution, while Cu concentrations increased linearly.

The results are useful for improving the quality of the honey value chain. An integrated program on quality assurance should be conducted. Beekeepers should pay attention to the location of the apiaries since there still are high accumulations of heavy metals in the area. Because of the interrelation between environmental pollution and food, honey and bee products need to be carefully monitored to eliminate even suspected contamination, especially in a historically polluted area from appointed source pollution, such as the town of Copșa Mică and its neighborhood. This study also has several limitations related to the limited number of analyzed apiaries and research areas. For future studies, the research area should be extended, and comparative analysis should be conducted.

## Figures and Tables

**Table 1:** Location of the apiaries.

No.	Plot Identification	Site Description in Relation with Pollution Edge	Distance from the Main Pollution Source (km)
1	46°03′27.97″ N, 24°04′28.43″ E, 312 m.a.s.l	Secondary valley channeling the atmospheric circulation of pollutants from the main valley	9.90
2	46°05′10.60″ N, 24°05′29.97″ E, 283 m.a.s.l	Site frontal exposure to thepollution source	10.54
3	46°05′24.03″ N, 24°06′52.86″ E, 280 m.a.s.l	Site frontal exposure to thepollution source	8.72
4	46°08′17.88″ N, 24°17′26.16″ E, 287 m.a.s.l	Site frontal exposure to thepollution source	5.91
5	46°05′03.91″ N, 24°16′30.08″ E, 340 m.a.s.l	Secondary valley channeling the atmospheric circulation of pollutants from the main valley	5.49
6	46°06′23.92″ N, 24°13′27.14″ E, 310 m.a.s.l	Site frontal exposure to thepollution source	1.12

**Table 2:** Temperature program of digestion.

Step	1	2	3	4
Temp. [°C]	145	170	190	100
Ramp [min]	2	5	2	1
Hold [min]	5	10	15	10

**Table 3:** Instrumental analytical values of investigated trace elements in FAAS.

Element	Flame Type	Fuel FlowL/min	Lamp Current mA	Wavelength nm	Slit Width nm	Air Flow L/min
Zn	Air-Acetylene	2.00	3	213.9	0.7	17.00
Cu	Air-Acetylene	2.00	3	324.8	0.7	17.00

**Table 4:** GFAAS program for non-essential trace elements Pb and Cd.

Non-Essential Trace Metal	Pb	Cd
Wavelength (nm)	283.3	228.8
Argon flow (Ml/min)	250	250
Sample volume (µL)	20	20
**Heating Program Temperature (°C; ramp time (s), hold time (s).**		
Drying 1	100 (5.20)	100 (5.20)
Drying 2	140 (15.5)	140 (15.15)
Ashing	700 (10.20)	700 (10.20)
Atomization	1800 (0.5)	1650 (0.5)
Cleaning	2600 (1.3)	2600 (1.3)

**Table 5:** Statistical data on heavy metal content in polyfloral honey.

Metal	Range	Median	Coefficient of Variation (%)	The Significance of the Differences between Plots	
				*t*	*p*
Pb (mg/kg)	0.76–3.41	1.49	56.27	4.35	0.007
Cd (mg/kg)	0.05–3.81	2.20	81.90	2.99	0.030
Zn (mg/kg)	15.00–36.40	20.40	36.74	6.67	0.001
Cu (mg/kg)	2.00–33.00	3.70	122.63	2.00	0.100

**Table 6:** Stratification of heavy metal concentrations in polyfloral honey.

Dependent Variable				
Grouping variable	Pb	Cd	Zn	Cu
*p* from Mann–Whitney U test (0.05 is the threshold value for statistical significance)				
Exposure to air circulation	0.05	0.64	0.64	0.90

**Table 7:** Comparative results on honey contamination with Zn and Cu in different research areas.

Literature Source	Zn Concentration (mg/kg)	Cu Concentration (mg/kg)	Honey Samples Source
Dzugan et al., 2017 [60]	12.57	0.77	Polyfloral honey/Podkarpackie southeastern Poland
Derebasi et al., 2014 [59]	0.15–0.17	0.17–0.19	Turkey, Black Sea Region
Tuzen and Soylak, 2005 [26]	1.1–24.2	0.25–1.10	Turkey, Central Anatolia Region
Berinde and Michnea, 2013 [52]	1.09–1.39	0.24–0.32	Romania, city of Baia Mare
Bratu and Georgescu, 2005 [62]	2.3		Honey samples provided by Bee Breeders Association/unpolluted area
	1.8–5.6		Romania, CopșaMică, polluted area 8–25 km away from the source of pollution
Ciobanu and Rădulescu, 2016 [65]	0.987	18.89	Polyfloral honey/Romania, Timiș County, in the vicinity of sources of pollution
Celechovska and Vorlova, 2001 [57]	0.190–22.9	0.057–1.55	Honey samples from the Czech market
Devillers et al., 2002 [63]	0.04–5.96	0.03–2.30	Acacia honey/Polluted and unpolluted areas of France
Roman et al., 2011 [53]	0.51–7.85	0.45–2.43	Poland, Wroclaw
Matusevicius et al., 2010 [58]	0.564–5.008	0.1106–0.3894	Honey from various areas of Lithuania
